# Solving polymicrobial puzzles: evolutionary dynamics and future directions

**DOI:** 10.3389/fcimb.2023.1295063

**Published:** 2023-12-08

**Authors:** Abijith Srinivasan, Anusree Sajeevan, Shobana Rajaramon, Helma David, Adline Princy Solomon

**Affiliations:** Quorum Sensing Laboratory, Centre for Research in Infectious Diseases (CRID), School of Chemical and Biotechnology, SASTRA Deemed to be University, Thanjavur, India

**Keywords:** ecology, microbial interaction, quorum sensing, game theory, eco-evolutionary dynamics

## Abstract

Polymicrobial infections include various microorganisms, often necessitating different treatment methods than a monomicrobial infection. Scientists have been puzzled by the complex interactions within these communities for generations. The presence of specific microorganisms warrants a chronic infection and impacts crucial factors such as virulence and antibiotic susceptibility. Game theory is valuable for scenarios involving multiple decision-makers, but its relevance to polymicrobial infections is limited. Eco-evolutionary dynamics introduce causation for multiple proteomic interactions like metabolic syntropy and niche segregation. The review culminates both these giants to form evolutionary dynamics (ED). There is a significant amount of literature on inter-bacterial interactions that remain unsynchronised. Such raw data can only be moulded by analysing the ED involved. The review culminates the inter-bacterial interactions in multiple clinically relevant polymicrobial infections like chronic wounds, CAUTI, otitis media and dental carries. The data is further moulded with ED to analyse the niche colonisation of two notoriously competitive bacteria: *S.aureus* and *P.aeruginosa*. The review attempts to develop a future trajectory for polymicrobial research by following recent innovative strategies incorporating ED to curb polymicrobial infections.

## 1 Introduction

Infections commonly involve diverse microorganisms, with bacteria present initially but eventually being replaced by new species that establish colonization. The distinction between a commensal and a pathogenic bacterium arises from their co-evolution within the host (Ghosh, 2013). Bacteria that persist undergo genetic changes, such as immune evasion mechanisms, enhancing their fitness and leading to chronic infections that resist treatment. Therefore, understanding the microbial causes of chronic infections necessitates a focus on these pivotal “Game Changing” (GC) bacteria (Grant and Hung, 2013).

Two prominent GC bacteria that are often co-isolated from chronic infections are *Pseudomonas aeruginosa* and *Staphylococcus aureus.* They have been isolated from chronic infections like chronic wounds (Gjødsbøl et al., 2006), pulmonary cystic fibrosis (Henson et al., 2019), chronic osteomyelitis (Masters et al., 2022), and chronic otitis media (Viswanatha, 2017). The dyad, therefore, is of immense interest to researchers studying chronic polymicrobial infections. The dyad might also be a triad with the fungal pathogen *Candida albicans.* It has been known to play a significant role in similar chronic scenarios. Hence this review will also explore the interlink between fungi and bacteria. In cases of chronicity, the involved bacteria undergo mutations or trade-offs to acclimate to the environment, ensuring their persistence (Marvig et al., 2015). This fitness improvement is guided by eco-evolutionary dynamics and evolutionary game theory. The convergence of these two concepts gives rise to a relatively novel idea: eco-evolutionary game theory (Tilman et al., 2020; Mondal et al., 2022). These concepts are underscored in the review and categorized under evolutionary dynamics (ED). Moreover, it is worth noting that both eco-evolutionary dynamics and evolutionary game theory involve mathematical analysis. However, the review does not primarily delve into mathematical modeling; instead, it places greater emphasis on the exploration of the decision-making processes and their potential applications. It also integrates established research on these principles, interlinking them with validated models of co-existence observed in bacteria. Subsequently, these impactful principles are extrapolated to diverse domains within biological sciences, encompassing genetic engineering, synthetic biology, evolutionary entrapment, antimicrobial resistance (AMR), microbial biosensors, and investigations into the microbiome.

## 2 Infectome

The term “infectome” pertains to the assemblage of microbes an individual encounters during infection (Bogdanos et al., 2013). However, its direct relevance to the individual’s prognosis might not be absolute, given that the infectome might not precisely mirror the present bacterial composition. Noteworthy instances of chronic infections include chronic wounds, chronic pulmonary cystic fibrosis, chronic otitis media, chronic dental abscesses, chronic osteomyelitis, and Catheter-Associated Urinary Tract Infections (CAUTI), which serve as prominent models of polymicrobial infections. In this context, we explore the prevalence and attributes of the GC bacteria of various infections (**Table 1**).

**Table 1:** The infectome of persistent polymicrobial infections.

S.no	Disease	Consortia	Composition (%)	Reference
1	Chronic wounds	*Pseudomonas aeruginosa*	52.20	(Gjødsbøl et al., 2006)
		*Staphylococcus aureus*	93.50	
		*Enterococcus faecalis*	71.70	
		*coagulase-negative staphylococci*	45.70	
		*Proteus species*	41.30	
		anaerobes	39.10	
2	Chronic pulmonary cystic fibrosis	*Pseudomonas aeruginosa*	85.30	(Henson et al., 2019)
		*Staphylococcus aureus*	34.70	
		*Burkholderia capecia*	4	
		*Streptococcus sanguinis*	88	
		*Prevotella*	75	
		*Escherichia coli*	4	
3	Chronic oestyomyletes	*Staphylococcus aureus*	45-55	(Masters et al., 2022)
		*Streptococcus* spp.	5-20	
		*Pseudomonas spp*	10-20	
		*Enterobacteriaceae spp*	10-15	
		Fungal	<5	
4	Acute Otitis media (AOM)	*Moraxella Catherillis*	3-20	(Vergison, 2008)
		*Heamophillus influenzae*	80	
		*Streptococcus pneumoniae*	80	
		group A *Streptococcus*	1-5	
5	Chronic otitis media with effusion (COME)	*Alloiococcus otitidis*	44.10	(Korona-Glowniak et al., 2020)
		*Moraxella catarrhalis*	8.80	
		*Streptococcus pneumoniae*	13.20	
		*Haemophilus influenzae*	20.60	
6	Chronic supparative otitis media (CSOM)	*Pseudomonas aeruginosa*	37.21	(Viswanatha, 2017)
		*Staphylococcus aureus*	27.91	
		*klebsiella pneumoniae*	13.95	
		*Proteus spp*	10.46	
		*Streptococcus pneumoniae*	3.49	
		*Escherichia coli*	4.65	
		*Streptococcus pyogens*	2.33	
7	Dental carries	*Streptococcus mutans*	20	(Hoceini et al., 2016)
		*lactobacillus acidophilus*	22	
		*Enterococcus faecium*	38	
		*Actinomyces naeslundii*	30	
		*Candida albicans*	65	Raja et al., 2010
		*Candida tropicalis*	25	
		*Candida parapsilosis*	8.33	
8	Catheter-associated urinary tract infection (CAUTI)	*Escherichia coli*	23.90	Weiner et al., 2016
		*Candida albicans*	11.70	
		*Staphylococcus aureus*	1.60	
		*Pseudomonas aeruginosa*	10.30	
		*Klebsiella pneumoniae*	10.10	
		*Enterococcus feacalis*	7	

The prevalence of polymicrobial biofilms displays considerable variability influenced by a range of host-related environmental factors, leading to a dynamic state. Predominantly, the frequency of the chronic wound bacterial consortia is *Pseudomonas aeruginosa* (52.2%), *Staphylococcus aureus* (93.5%), *Enterococcus faecalis* (71.1%), coagulase-negative staphylococci (45.7%), *Proteus* spp. (41.3%), along with other anaerobic bacteria (39.1%)(Gjødsbøl et al., 2006). Moreover, chronic wounds also harbor fungi like *Candida albicans* and viral elements such as phages and viruses (Verbanic et al., 2022) making it a more complex microbial environment. *P. aeruginosa* decreases species diversity due to its highly predatory nature (Tipton et al., 2020) attributed to its virulence and quorum sensing. In such instances, a multitude of bacteria falls victim to the effects of exoproducts released by *P. aeruginosa*, ultimately resulting in their incapability to maintain their existence.

*Candida albicans* showcases cooperative behavior and establishes a stable community with *S. aureus*, whereas *P. aeruginosa* engages in competition, aiming to eliminate both *S. aureus* and *C. albicans* (Morales and Hogan, 2010). Within polymicrobial infections, fungal pathogens like *C. albicans* could potentially fortify coexistence because of their exoproducts (De Sordi and Mühlschlegel, 2009; Hassan Abdel-Rhman et al., 2015). Notably, farnesol, an exoproduct of *C. albicans*, is known to inhibit Pseudomonas Quinone Sensing (PQS), a crucial component responsible for bactericidal effects (Cugini et al., 2007). In addition, Pastar et al., have demonstrated that the virulence of *S. aureus* has been altered in the presence of *P. aeruginosa* polymicrobial biofilm (Pastar et al., 2013). The antibiotic susceptibility of bacteria in polymicrobial wounds is different from monomicrobial wounds (Sun et al., 2008). In the presence of *S. aureus*, *P. aeruginosa* prompts alterations in its Lipopolysaccharide (LPS) side chain, enhancing resistance against beta-lactam antibiotics (Tognon et al., 2017). Additionally, *S. aureus* has been observed to generate Small Colony Variants (SCVs) when coexisting with *P. aeruginosa*, potentially contributing to recurrent infections (Melter and Radojevič, 2010).

An intriguing phenomenon observed involves an increase in the concentration of anaerobic populations as chronic wounds progress and mature. Nevertheless, studying these bacteria is complex because of their unique characteristics, and it requires a more comprehensive exploration (Percival et al., 2018). Anaerobes play a pivotal role in sustaining coexistence by influencing critical oxygen tensions that impact mutagenesis. Notably, *P. aeruginosa* leverages anaerobic conditions as a cue for developing chronic infections, triggering amplified biofilm formation while curtailing dispersion (Cao et al., 2023). *Acinetobacter baumannii* and *Staphylococcus epidermis* have been known to be causative agents for multiple nosocomial infections. *A. baumannii* is a known polymicrobial constituent in upper respiratory tract infections and has been commonly co-isolated with *P. aeruginosa* and *K. pneumoniae* (Gedefie et al., 2021). It is a red-priority pathogen as per WHO guidelines and forms robust biofilms in air-liquid interface compared to other *Acinetobacter* spp (Cerqueira and Peleg, 2011). Additionally, it is recognized for its ability to endure in various environmental niches and possesses a remarkable degree of genomic plasticity (Eze et al., 2018).

**Figure 1:**
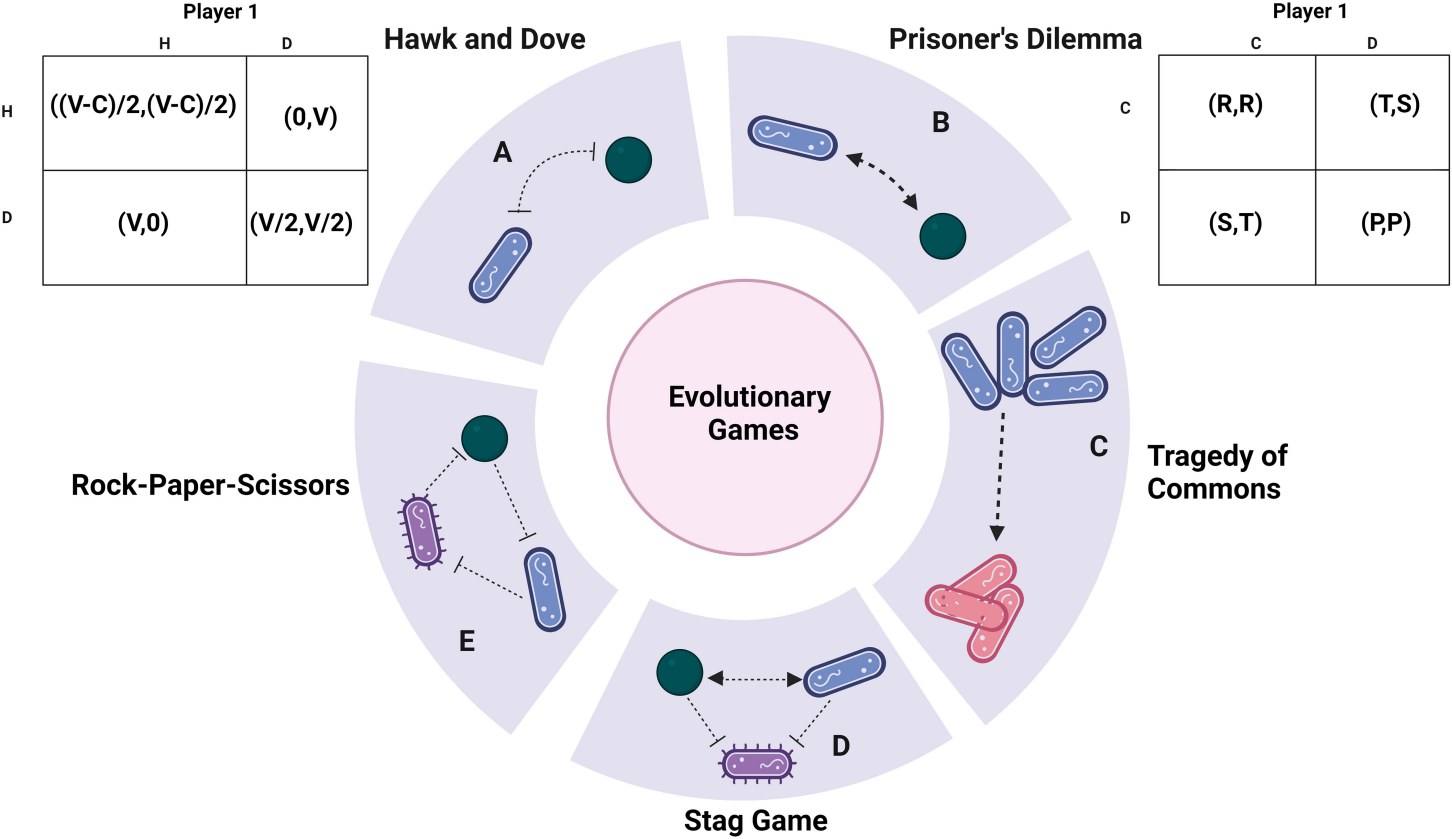
Payoff analysis: **(A)** The Hawk and Dove game theory supports aggressive interactions between bacteria. The associated pay-off matrices are expressed as (X, Y), where X represents the pay-off for bacteria 1 and Y corresponds to bacteria 2. V represents the environmental resource, while C denotes the fitness cost due to competition. **(B)** The Prisoner’s Dilemma model, when iterated, serves as evidence of altruism. This model revolves around cooperative behaviour. **(C)** The Tragedy of the Commons game theory simulates the scenario of cheating. The arrow here highlights how cheaters gain a fitness advantage, invade a population and subsequently change attain an equilibrium. Pink refers to cheaters while blue are wild type cells. **(D)** The Stag game demonstrates the cooperative behaviour of uniting against a common adversary. It portrays the collaboration between hunter bacteria and the competition against prey bacteria. **(E)** The Rock-paper-scissors game theory showcases a captivating dynamic equilibrium among bacteria. The arrowheads refer co-operative tendency while inhibitor arrows show aggression unless specified. Created with BioRender.com.

*Enterococcus faecalis* emerges as another frequently co-isolated bacterium in both chronic wounds and CAUTI (**Table 1**). The bacterium is known to actively manipulate the environmental pH, creating an acidic environment that inhibits the growth of *P. aeruginosa* (Tan et al., 2022). Additionally, it enhances the survival of *S. aureus* through metabolic synergy, as illustrated in **Figure 1** to recurrent infections. The striking similarity between the persistent microorganisms found in both chronic wounds and cystic fibrosis prompts the consideration that shared host immune interactions could also play a role. A molecule of paramount importance in this context is calprotectin, a widely distributed entity that imparts nutritional immunity (Murdoch and Skaar, 2022). Notably, calprotectin is recognized for its iron chelation properties, which curtail the fitness benefits of bacteria and contribute to persistence (Wakeman et al., 2016).

Two clinically relevant GC bacteria for soft skin infections are *Staphylococcus epidermis* and *S. aureus* (Chen et al., 2018). *S. epidermis* is known to hinder biofilm formation in *S. aureus* through the action of Esp serine protease. However, it’s important to note that this inhibition doesn’t compromise the survival of Staphylococcus aureus (Fredheim et al., 2015; (Kohda et al., 2021). Another microorganism frequently found alongside *S. epidermis* in nosocomial infections is *Candida tropicalis*. Both these organisms have the propensity to create polymicrobial biofilms implicated in cases of bloodstream infections, with episodes of septicemia preceding candidemia (Bouza et al., 2013; Kim et al., 2013). Similar to *S. aureus*, *C. tropicalis* has also been shown to improve the biofilm formation of *S. epidermis* using farnesol. Conversely, *S. epidermis* enhances hyphal invasion by attaching to hyphal (Phuengmaung et al., 2021).

Chronic osteomyelitis presents an intriguing scenario wherein *S. aureus* persistence predominantly prevails across most infections, unlike the prevalence of *P. aeruginosa* in cystic fibrosis (Jerzy and Francis, 2018; Huang et al., 2022). Jerzy et al. also show the immense ability of resistance to antibiotics, specifically methicillin, commonly referred to as MRSA (Methicillin-resistant *S. aureus*), which also showed high sensitivity to ciprofloxacin, erythromycin, and gentamycin. These effects are attributed to the lower oxygen content maximizing resistant phenotypes in bacteria. An intriguing aspect involves the concept of collateral sensitivity, manifesting between aminoglycosides and beta-lactam antibiotics (Lozano‐Huntelman et al., 2020). Collateral sensitivity refers to the evolutionary trade-off interaction wherein resistance to one antibiotic translates into susceptibility to another. This dynamic interaction is intricately guided by eco-evolutionary dynamics.

A chronic variant of otitis media manifests in two distinct modes: chronic otitis media with effusion (COME) and chronic suppurative otitis media (CSOM) (Niehus et al., 2021). COME is characterized by fluid accumulation behind the tympanic membrane, while CSOM involves the persistent rupture of the tympanic membrane with ongoing otorrhoea or discharge of pus. The microbiological profile of CSOM reveals the presence of MRSA, *P. aeruginosa*, and other pathogens such as *Alloicoccus* and *Streptococcus* (Neeff et al., 2016). Neef and colleagues contend that the abundance of *P. aeruginosa* was noticeably reduced compared to the findings reported in earlier studies (Mittal et al., 2015). The disparity between molecular and culture-based techniques and the importance of this pathogen has not been understood in the context of CSOM. Reliable induction of CSOM in mice requires perforation of the tympanic membrane along with blockage of the eustachian tube with anti-healing agents or the usage of bacteria like *Streptococcus pneumoniae* or *P. aeruginosa* (Bhutta et al., 2017).

Dental caries presents yet another prevalent example of a chronic polymicrobial infection. The condition is primarily dominated by cariogenic bacteria that metabolize sugars, resulting in acid production (Wu and Ross, 2016). The aciduric bacteria thrive within the altered microenvironment, displacing commensal microorganisms and instigating the process of demineralization (Selwitz et al., 2007). This phenomenon is referred to as the ecological plaque hypothesis. Unlike the earlier notions of specific and non-specific plaque hypotheses that centre around the bacteria themselves, the ecological plaque hypothesis focuses on the community’s ecology influenced by the surrounding environment (Lambert et al., 2014). Thus, forms a foundational element of eco-evolutionary dynamics, contributing to the rationale behind selecting this infection as a model.

## 3 Evolutionary game theory

Standard game theory was designed to analyze social dilemmas using mathematical modeling. A game entails multiple players/participants that utilize strategies to improve their payoff and settle upon a result. In bacterial populations, this pay-off can be attributed to fitness. Most evolutionary games lead to an evolutionary stable strategy (ESS), the best possible payoff for both the players. Evolutionary Game Theory (EGT) predicts the stability of mutants that compete over resources with the wild type through a variety of social dilemmas. As a result, it directly correlates with the evolutionary fitness and persistence of bacteria. EGT encompasses a range of mathematical models for dissecting logical scenarios, as elaborated below. EGT, a mathematical model used for evaluating the Evolutionarily Stable Strategy (ESS), and remains true irrespective of the organism unless the logicality of the scenario (primarily competition) (Lambert et al., 2014). The investigation of cooperation within a community is a subsequent exploration, which delves into eco-evolutionary dynamics and the concept of metabolic syntropy.

EGT is commonly applied to comprehend the dynamics of mutant coexistence and invasion within populations, as well as the intricacies of co-evolution in multi-player scenarios (Adami et al., 2016). Notably, it’s imperative to recognize that mathematical modelling plays a pivotal role in elucidating the presence of Evolutionary Stable Strategies (ESSs) specific to a given scenario. Although the endurance or adherence to these ESSs might not be constant, they establish fundamental principles for logical interactions and decision-making. EGT analysis and mathematical modelling are conducted within a predetermined environment, solely considering inter-specific resource competition among bacteria. Consequently, these approaches do not delve into how new environments might further impact evolution/fitness or how other inter-specific interactions could influence the prognosis. Furthermore, EGT does not examine the ramifications of these ESSs or the logical decisions they entail, aspects of paramount significance in the context of infections.

### 3.1 Hawk and dove game

The classic “Hawk and Dove” game, also referred to as the “snowdrifter’s game,” presents a scenario where hawks and doves engage in competition over a limited resource (Smith and Price, 1973). In this context, the hawk-type bacteria are characterized as highly competitive and aggressive, while the doves adopt a more peaceful approach, evading threats. This social dilemma framework has previously been employed to assess the persistence of *Klebsiella pneumoniae* and *P. aeruginosa*, exemplifying its relevance in analyzing inter-specific competition and persistence within polymicrobial settings (Gergely, 2022). The payoff matrix (**Figure 1**) visually depicts the fitness of each bacterium in the presence of the other.

In confrontations between two hawks, the resulting net pay-off for each is calculated as (V-C)/2, with V representing the resource and C denoting the cost of combat. Conversely, when two doves contend, the resource is shared, yielding a net payoff of V/2. When a hawk engages a dove, it claims all available resources, while a dove confronting a hawk loses its entire resource pool. An Evolutionarily Stable Strategy (ESS) materializes when the two bacteria adopt distinct strategies (one resembling a hawk and the other a dove). The framework lies at the core of competition sensing and bacterial interactions. The classical hawk and dove game, a symmetrical model, elucidates the evolution of aggression. When the cost of fighting (C) is less than the resource value (V), as typically occurs during the initial stages of infection, aggression consistently prevails(Sirot, 2000; Hsu et al., 2005; Kokko et al., 2014). Conversely, if the cost of fighting (C) exceeds the resource value (V) at any point, a mixed strategy becomes more favorable.

The social dilemma offers a clear insight: when a bacterium faces the threat of a predatory or dove-type bacterium, it is inclined to evolve offensive tactics as a countermeasure (Niehus et al., 2021). Niehus et al., employ EGT to demonstrate that the escalation of virulence can occur in response to stress signals emitted by “kin” cells that have been destroyed. Typically, such inter-specific competition arises prior to the establishment of persistence. Bacteria begin to evolve mechanisms aimed at enduring and conserving resources to survive prolonged periods of infection. The achievement is primarily facilitated through a spectrum of mechanisms, which encompass metabolite syntropy, niche localization, polymicrobial biofilms, and genetic mutations(Leinweber et al., 2017). An “N” player Hawk and Dove game (Chen et al., 2017) utilizes a dove threshold T, which shifts the equilibrium from hawk domination to dove domination depending on its value. The model also connects this to ecosystems where “doves” join to repel “hawks”. This is of immense interest as the commensal population (which are usually the doves) that infiltrate the location first are given time to improve their biomass. The snowdrift game can be successfully used to model interactions between pathogens and commensals (Wu and Ross, 2016). The type of interaction is highly common and valued in polymicrobial infections due to the primary colonizers being commensals. The Hawk and Dove game thus stands as a highly pertinent model within the realm of polymicrobial research, yet it remains relatively underexplored.

### 3.2 Prisoner’s dilemma

The “Prisoner’s Dilemma” stands as another iconic game frequently employed to elucidate altruistic behaviours. The widely known social dilemma revolves around two potential prisoners. These individuals, suspected of a more significant offense, are subjected to separate interrogations. If both prisoners disavow any involvement in the crime, both will receive the initial sentence of one year. However, if one prisoner confesses while the other remains silent, the confessor will receive a reduced original sentence, while the non-confessor will bear the weight of the more severe offense. Alternatively, if both prisoners confess, they will each face a lesser sentence, albeit for the more substantial crime (Smith and Schuster, 2019).

**Figure 2:**
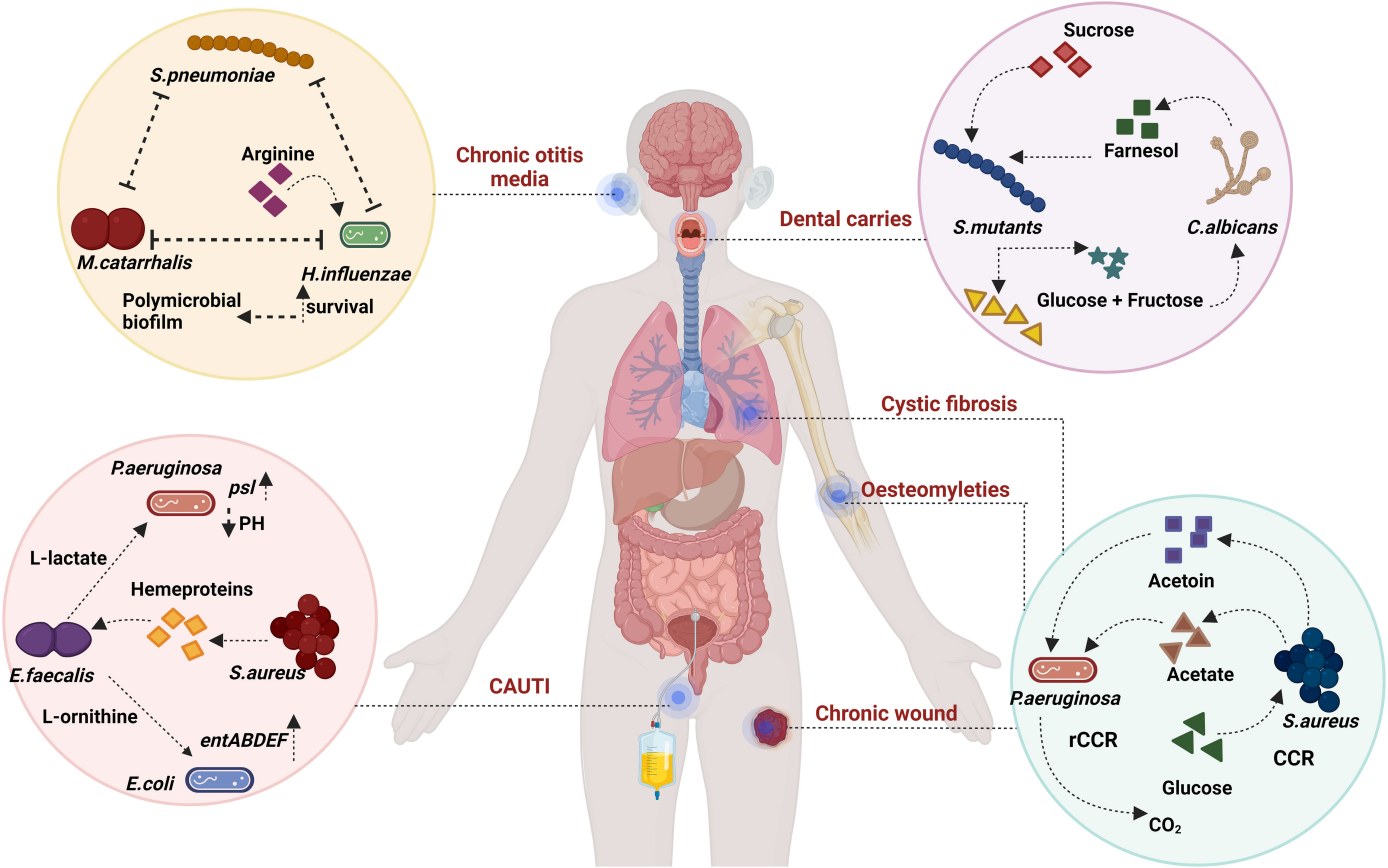
Metabolic syntropy: *S. mutans* and *C. albicans* exhibit metabolic interconnections within dental caries. In the context of *P. aeruginosa* and *S. aureus*, there is a general co-existence model involving rCCR and CCR bacteria. Within CAUTI, *E. faecalis* participates in cross-feeding interactions with both *E. coli* and *S. aureus*. *E. faecalis* also utilises nutritional immunity to induce stress and affect *P. aeruginosa*. Additionally, an analogy to the rock-paper-scissors game emerges among *S. pneumoniae*, *M. catarrhalis*, and *H. influenzae*, where the availability of arginine plays a crucial role in their co-existence dynamics. Created with BioRender.com.

A payoff matrix (**Figure 2**) is formulated for this game, with the payoff values ranked as P<R<S<T. The optimal payoff can be achieved if the player consistently chooses to confess, resulting in either R or S years of imprisonment. The most favourable potential outcome involves the prisoners denying the accusation, leading to only P years in prison. However, there’s a risk associated with this choice, as it hinges on the cooperation of the partner, and failure to cooperate could result in T years of imprisonment. This illustrates a preference for non-cooperative behaviour over cooperation (Lambert et al., 2014; Smith and Schuster, 2019). This phenomenon is quite common, as resorting to aggression is often considered the ‘safer’ course of action in challenging situations, even though cooperation might yield superior outcomes. In a scenario where the game is repeated multiple times, the players tend to adjust their strategies over time. In an iterated Prisoner’s Dilemma, cooperation is favoured through strategies like the ‘tit-for-tat’ approach, where actions mirror the partner’s previous move (Axelrod and Dion, 1988; El-Seidy and Soliman, 2016).

### 3.3 Tragedy of the commons

Unveiling the “Tragedy of the Commons”: In this scenario, we encounter a frequently observed social paradox, where non-cooperation prevails even amid shared resources. The phenomenon finds a parallel in the realm of polymicrobial infections, where an individual bacterium might strategically monopolize resources to augment its own fitness. Drawing inspiration from the notion of water scarcity, envision two families bestowed with a plenteous yet finite water reservoir. Paradoxically, both families tend to indulge in excessive water consumption, overlooking the potential dividends of measured usage. Strikingly, the optimal resolution springs forth when both families opt for judicious water utilization. The reluctance for cooperation stems from the understanding that prudent resource allocation by one party can become a detriment to the overall health of the collective. Within the intricate backdrop of bacterial interactions, the accumulation of resources, exemplified by quorum sensing secretions (Özkaya et al., 2018; Feng et al., 2019; Gergely, 2022), can propel the emergence of genetic mutants that exploit these resources without reciprocating, known as the intriguing “cheater” phenomenon.

The recurring response is firmly anchored in situations characterized by limited resources, where shared exoproducts can serve as catalysts for the emergence and persistence of cheaters within the population (Chen et al., 2019). Cheaters serve as significant players in mitigating bacterial virulence (Rumbaugh et al., 2009). Their strategic role involves diverting the focus of bacterial biomass production away from the overproduction of exoproducts. For instance, in the context of *P. aeruginosa*, cheaters orchestrate intra-specific dynamics by embracing the division of labour, which subsequently stabilizes multiple sub-populations and evolutionary lineages (Özkaya et al., 2018). The strategic interplay resonates with the principles of the snowdrift’s game payoff matrix, notably due to the influence of producer diffusion rates on cheaters (Xenophontos et al., 2022). A dynamic coexistence between the wild type and the cheaters is maintained, as the cheaters are tied to the production of common goods by the wild type, for survival.

### 3.4 Rock paper scissors

The rock-paper-scissors dynamic represents a captivating three-player game characterized by cyclic coexistence among the bacterial participants. Specifically, Bacteria A exerts inhibition over Bacteria B, which reciprocally suppresses Bacteria C. In the continuation of this cyclic interaction, Bacteria C inhibits Bacteria A. The intricate interplay yields a spectrum of complex and intriguing outcomes. One notable phenomenon is the “survival of the weakest (Frean and Abraham, 2001), wherein the inhibition of a particular bacterium triggers an imbalance that fosters the resurgence of the previously inhibited species. So, underscores the intricate nature of addressing a singular bacterium within a polymicrobial infection, as such an approach might not yield viable outcomes.

Another intriguing aspect to consider is the emergence of altruism—a form of selfless behaviour that stands in contrast to cheating. However, such altruistic behaviour is often less favoured due to the vulnerability it exposes to exploitation by cheaters, a pattern similar to what is observed in other gaming scenarios. Within the cyclic dominance inherent in the rock-paper-scissors game, a form of restraint akin to altruism emerges in *E. coli* (Nahum et al., 2011). The game has unveiled the coexistence of *E. coli* strains producing colicin, both s and resistant strains, thus illustrating antibiotic antagonism (Kirkup and Riley, 2004), and rekindling the connection to the hawk and dove model. Additionally, allelopathy assumes a role in the dynamic equilibrium of colicin strains within *E. coli* (Durrett and Levin, 1997). The interplay between allelopathy and motility, intertwined dynamics, influences the biodiversity and persistence of bacterial communities (Kerr et al., 2002; Reichenbach et al., 2007). Thus, the phenomenon of allelopathy is elucidated further in the upcoming section. The effect of this game aligns with the border theme of genetic engineering and drug research is discussed in the applications. It might be necessary to develop pay-off matrices in the presence of multiple games with mutations to analyze situations more closely to nature (Roy et al., 2022) (**Figure 1**).

## 4 Community interactions

In the realm of inter-microbial interactions, a spectrum of dynamics is explored, encompassing mutualism, antagonism, commensalism, amensalism, and predation (Tshikantwa et al., 2018). Among these, mutualism stands out as a noteworthy phenomenon, denoting inter-specific cooperation that confers a net positive fitness to all participating organisms. In the bacterial context, this cooperative behaviour is manifest in the form of syntropy (Wintermute and Silver, 2010). Syntropy pertains to the mutual sharing or cross-feeding of metabolites between two or more bacterial entities. Such interactions have a profound impact on the fitness of bacterial sub-species engaged in syntropy, thereby gaining Favor through natural selection, and fostering interdependence, ultimately leading to the emergence of mutualism (Bshary and Grutter, 2002) (**Figure 2**).

In instances like acute otitis media, *M. catarrhalis*, *H. influenzae*, and *S. pneumoniae* dominate the bacterial landscape. *H. influenzae* and *S. pneumoniae*, through synergistic cooperation, create denser biofilms by manipulating multiple genes (Tikhomirova et al., 2015). While a stronger polymicrobial biofilm is generated in the presence of both bacteria, *S. pneumoniae* modifies *H. influenzae’s* lipopolysaccharide (LPS) coating, rendering it more susceptible to host defences. Apart from desialylation of the cell surfaces, *S. pneumoniae* also utilise hydrogen peroxide to inhibit other bacteria *in vitro* (Shakhnovich et al., 2002). Conversely, *H. influenzae* enhances opsonophagocytosis against *S. pneumoniae* (Lysenko et al., 2005). Additionally, it has been demonstrated that these interactions depend on pH and nutrients like arginine (Tikhomirova et al., 2015; Tikhomirova et al., 2018). Arginine is involved in enhancing ATP production in *H.influenzae*, using the arginine deaminase pathway. Tikhomirova et al. show that in a triple species co-culture, there is a competition that avails between all three bacteria (Tikhomirova et al., 2022). These interactions are also shown to be strain-specific and time-point-specific. The authors also hypothesise that arginine upregulation is a mechanism of survival during conflict and is exclusively used by *H. influenzae* in co-cultures as opposed to monocultures (Tikhomirova et al., 2022). The authors also show that ATP production in *H. influenzae* also supplements ATP production in *M. catarrhalis* and *S. pneumoniae*. Further exogenous addition of arginine did not confer an improvement of ATP. This along with the fact that all three bacteria lack the ability to metabolize arginine leads to the authors arguing the relevance of arginine, a nutrient for maintaining this polymicrobial consortia at early stages of infection.

A bi-directional persistence mechanism is present in dental carries between *S. mutans* and *C. albicans* (Kim et al., 2017). Farnesol, an antibacterial compound produced by *C. albicans*, has been demonstrated by Kim et al. to support *S. mutans* biofilm growth at low concentrations. The coexistence of these organisms is pivotal in niche colonization and pathogenesis*. S. mutans*, adept at degrading sucrose into glucose and fructose, contributes an energy source that *C. albicans* can utilize, as the latter cannot break down sucrose (Sztajer et al., 2014). The interaction further activates various genes involved in extracellular polysaccharide synthesis, competence, glucosyltransferase (Gtf) production, and virulence (Falsetta et al., 2014). Cross-kingdom biofilm formation occurs due to the strong binding affinity between O-mannan of *C. albicans* and GtfB of *S. mutans* (Hwang et al., 2017).

*P. aeruginosa* and *S. aureus* also share a metabolic syntropy in CF patients. *S. aureus* secretes acetoin which is shown to activate *aco* (Acetoin catabolic pathway) genes in *P. aeruginosa* (Camus et al., 2020). Camus et al., hypothesize that accumulation of acetoin leads to the downregulation of acetoin metabolism further leading to the death of *S. aureu*s (Camus et al., 2020). *P. aeruginosa* and *S. aureus* in chronic wounds may persist through a cooperative division of labour (Park et al., 2020). Park et al., further develop a model of bistability between CCR (carbon catabolite repression) and rCCR (reverse carbon catabolite repression) bacteria (Park et al., 2020). CCR bacteria prefer to utilize carbon sources like glucose to generate exoproducts like organic acids whereas rCCR bacteria utilize organic acids to produce CO_2._ In the model, *P. aeruginosa* takes the role of rCCR bacteria which catabolizes acetate, a toxic byproduct of *S. aureus* secreted via glucose metabolism. It should also be noted that catabolite repression control (CbrA, a two-component system) is very important in generating *lasR* mutants in *P. aeruginosa* (Mould et al., 2022). The cross-feeding may play a huge role in why LOF (Loss of function) mutants are not formed in cocultures of *P. aeruginosa* (Luján et al., 2022). CbrA through the regulator CbrB, focuses on carbon source utilization like succinate over other sources like amino acids (Sonnleitner et al., 2009).

CAUTI is a serious polymicrobial infection that has a high chance of developing long-term catheterization (>30 days) (Parida and Mishra, 2013). *E. faecalis* from CAUTI has syntropy with *S. aureus* via heme proteins (Ch’ng et al., 2022). *E. faecalis* cannot secrete heme and hence only exogenously acquires it (Baureder and Hederstedt, 2012). Ch’ng et al., show that the secreted heme proteins of *S. aureus* can cross-feed *E. faecalis* with active gelatinase activity. Another vital interaction is between the most common bacteria *E.coli* (**Table 1**) and *E. faecalis*. *E. faecalis* has the ability to augment the biofilm of *E. coli* and induce the expression of enterobactin genes via L-ornithine (Keogh et al., 2016). Keogh et al., illustrate this phenomenon can occur during iron-limiting conditions, which are commonly encountered *in vivo. E. faecalis* secretes a small protein, EntV which has the capability to downregulate virulence and hyphal morphogenesis in *C. albicans* (Graham et al., 2017). *E. faecalis* has also shown the ability to improve *psl* polysaccharide production in *P. aeruginosa* (Lee et al., 2017) similar to *S. aureus* inducing *psl* production (Chew et al., 2014) which leads to a denser biofilm. Lee et al., claim that this interaction may be common in *P. aeruginosa* and gram-positive coccus. *E. faecalis ldh1* gene metabolises L-lactate through lactate dehydrogenase pathway. Due to iron-limiting condition and L-lactate’s chelation properties it affects nutritional and survival capability of *P. aeruginosa* inducing immense stress (Tan et al., 2022).

Nosocomial infections also involve multiple bacteria engaging in cross feeding mechanisms. *A. baumannii* engages in cross feeding with *K. pneumoniae*, where it metabolises fermentation sugars of the latter to form stronger biofilms than their monomicrobial counterparts (Semenec et al., 2023). Siderophores were downregulated in *A. baumannii* under co-culture conditions. Semenec et al., attribute this to siderophore sharing, where the wild type producing siderophores get outcompeted by cheaters that trade-off the costly production. This is another instance of ‘The tragedy of commons’ at play in polymicrobial biofilms.

According to the hawk and dove model, all bacteria engage in competition, either resource-mediated or bacteriocin mediated. Some bacteria are also involved in syntropy enhancing their ability to survive such competition, which is still mediated by the environment (Pajon et al., 2023). Therefore, natural selection favours these bacteria with altered metabolic interdependencies, leading to persistent polymicrobial illnesses (Henson et al., 2019). Such metabolic interdependencies can also be modeled using game theory (Zomorrodi and Segrè, 2017) (**Figure 2**).

## 5 Eco-evolutionary dynamics

Eco-evolutionary dynamics underscore the ongoing competition among bacteria, wherein the role of exoproducts and virulence factors as mediators varies. Bacteria exhibit specific pH tolerances and range that promote their thriving (Percival et al., 2014; Mougi, 2023). Their endeavour to modulate environmental pH aligns with these optimal ranges, and instances where interactions lead to a pH equilibrium result in minimal impact on fitness, thereby fostering persistence. This emphasizes the pivotal role of environmental feedback in comprehending bacterial decision-making. Microbial volatiles play a significant role in facilitating mediated eco-evolutionary dynamics within communities (Tyc et al., 2017), 88, (Jones et al., 2019). Additionally, bacterial membrane vesicles (BMVs) have emerged as contributors to eco-evolutionary feedback (Zlatkov et al., 2021).

EGT offers a robust framework for analyzing fitness perspectives and probabilities, but it remains highly abstract, providing a broad foundation for decision-making. However, the utility of such decision-making processes is contingent upon the examination of their impact on the environment and subsequent fitness outcomes. This underscores the necessity of combining eco-evolutionary dynamics (EED) with EGT. A suitable analogy for this amalgamation could be the relationship between a doctor and a pharmacist. A doctor’s prescription holds value only if the patient comprehends it, which is where pharmacists play a pivotal role. Pharmacists can dispense over-the-counter medications for minor ailments, yet they require a doctor’s expertise for complex disorders. Similarly, while game theoreticians may heavily concentrate on mathematical modeling, this approach sometimes sidelines experimental exploration (Traulsen and Glynatsi, 2023). This dynamic interaction between game theoreticians and evolutionary biologists is noteworthy.

While earlier research focused on how ecology shapes evolution or eco-evo interactions, the realization has emerged that evo-eco dynamics also play a significant role in ecosystems. The convergence of these two pathways gives rise to a novel bidirectional interaction known as eco-evolutionary feedback (EEF). This field of inquiry has gained momentum only recently, garnering the attention it deserves. According to a study, this feedback loop is predominantly implicated in microbial interactions, particularly within the context of polymicrobial diseases (Mougi, 2023). These feedback mechanisms contribute to our comprehension of bacterial interactions within polymicrobial illnesses, complementing the insights provided by EGT.

## 6 Quorum sensing mediated biofilm and virulence

Quorum sensing (QS) is a bacterial communication process that involves signal molecules (such as AIP, autoinducing peptide for gram-positive bacteria, and AHL, acyl homoserine lactone for gram-negative bacteria) (Miller and Bassler, 2001). In various contexts, polymicrobial interactions are intricately linked with quorum sensing, as these signaling circuits play a significant role in influencing metabolism (Zhao et al., 2019).

### 6.1 Quorum sensing system and virulence of *S.aureus*

*Staphylococcus aureus* uses a QS system *agr* (accessory gene regulator) which is acted by *sar* (staphylococcal accessory regulator) which is the global regulator. The signal is a peptide with thiolactone called AIP (autoinducing peptide). The *agr* locus has 2 units RNAII and RNAIII with P2 and P3 as their promoters respectively. RNAII possesses 4 genes *agrB, D, C*, and *A*. The gene *agrB* codes a transmembrane endopeptidase that induces the thiolactone modification in AIP (Tipton et al., 2020). Furthermore, the *agrC* and *agrA* genes encode a two-component signal transduction system. In this context, *agrC* functions as a histidine kinase sensor, while *agrA* serves as the corresponding response regulator. The downregulation of *agr* QS circuit expression encourages colonization and facilitates the expression of surface proteins. Conversely, higher expression leads to the generation of various toxins and virulence factors (Saenz et al., 2000). These factors encompass leucocidins, CHIPS (Chemotaxis inhibitory protein), and PSM (phenol soluble modulins). Leucocidins target the CCR5 chemokine receptor, lysing leukocytes and impairing the immune response (Alonzo et al., 2013). CHIPS reduces neutrophil recruitment to C5a complement, thus aiding immune evasion (de Haas et al., 2004). PSMs have the capacity to lyse macrophages, osteoblasts, erythrocytes, and neutrophils (Rasigade et al., 2013). Predominantly controlled by QS, SpA (Staphylococcal protein A) is a prominent surface protein with multifaceted functions. Its influence extends to inactivating the host’s humoral response (Becker et al., 2014). Other surface-binding proteins and virulence factors include coagulases and Von-Willebrand factor-binding proteins. These components aid the bacterium in evading immune responses by altering the coagulation cascade and binding to fibronectin-like extracellular matrix (ECM) proteins, respectively (McAdow et al., 2012).

### 6.2 Quorum sensing system and virulence of *P. aeruginosa*

*Pseudomonas aeruginosa* exhibits three primary quorum sensing circuits: the global regulator lasI/R, and two additional systems, rhlI/R and PQS. The auto-inducing signals include N-(3-oxododecanoyl)-L-homoserine lactone (3O-C12-HSL) for Las, N-butyryl-L-homoserine lactone (C4-HSL) for Rhl, and 2-heptyl-3 hydroxy-4-quinone for PQS (Pseudomonas quinone signal) (Pesci et al., 1999). Despite lasI/R being considered the global regulator, evidence suggests that rhlI/R can function independently (Wang et al., 2018). Notably, Wang et al. demonstrated a reduction in LasB elastase and minor pyocyanin production even when lasR is mutated. The Las operon encodes critical virulence factors like elastase, protease, pyocyanin, HCN, and rhamnolipids, many of which target and kill S. aureus (Filkins et al., 2015). Pyocyanin produces H_2_O_2_ and O^2-^ super radical by cycling itself in the presence of molecular oxygen (Mavrodi et al., 2001) during its stationary phase. Pyocyanin production depends on rhl and las pathways but recently it has been discovered to be produced in lasR mutants. Rhamnolipids are known to disperse biofilms and indulge in killing other microbial species (Wood et al., 2018). The elastases (LasA, Staphylolysin, LasB, Pseudo Lysin) have proteolytic activity and are also capable of inhibiting the growth of *S. aureus* (Kessler and Safrin, 2014). A novel quorum sensing circuit, integrated quorum sensing (IQS), has been identified, tightly regulated by las QS under normal conditions. Enhanced IQS expression triggers downstream factors through rhl and PQS circuits, especially under low phosphate conditions (Wood et al., 2018). There has been some controversy regarding whether IQS, the 4th quorum sensing molecule is produced from ambABCDE or from the pyocyanin siderophore biosynthesis pathway (Lee et al., 2013). Quorum sensing in *P. aeruginosa* is one of the reasons for the highly predatory nature of this bacteria. Multiple exoproducts of *P.aeruginosa* engage in chemical warfare.

*P. aeruginosa* utilizes type VI secretion systems (T6SS) to release antibacterial toxins (Hersch et al., 2020). While this secretion system has a very low success rate against gram-positive bacteria. T6SS has been shown to help in the uptake of nutrients like iron and manganese (Gallique et al., 2017) and repressed by las quorum sensing (Lesic et al., 2009). This could also play a huge role in the co-existence of gram-positive and gram-negative bacteria which is further explored in models of co-existence. Another interesting potential for coexistence is AHL crosstalk, this interaction is relatively newly explored between gram-negative bacteria (Wellington and Greenberg, 2019) (Gerdt et al., 2017). Similarly, type III secretion systems and type II secretion systems are also tied with quorum sensing (Pena et al., 2019). While T3SS is negatively regulated by las quorum sensing, T2SS is positively regulated by PQS quorum sensing. In addition, luxS, is a quorum sensing system that has known to be involved in inter-species communication (McNab and Lamont, 2003). Further peptidoglycans can also play a role in inter-kingdom signaling (Dworkin, 2014). Competition sensing is a process where stress responses are triggered under cellular damage and activate gene-encoding bacteriocins for a counterattack (Niehus et al., 2021).

## 7 Niche partitioning

**Figure 3:**
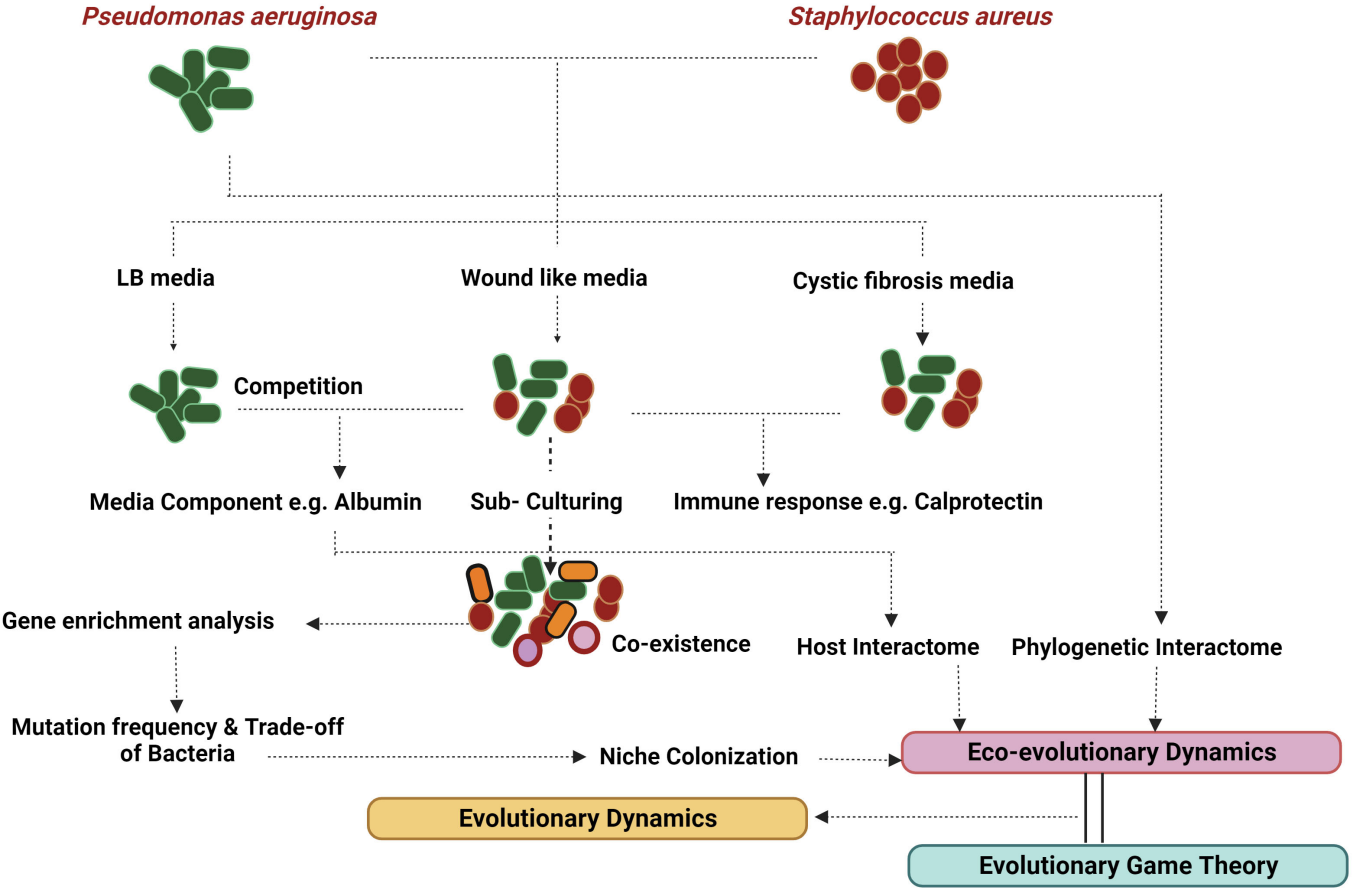
Evolutionary dynamics: Evolutionary dynamics encompasses a range of factors that contribute to fitness, including both eco-evolutionary dynamics and evolutionary game theory. *P. aeruginosa* and *S. aureus* have been shown to co-exist in CF and wound-like media but not in LB. This persistence can be modelled by analysis of the host factors and the phylogenetic radiation of the bacteria. Host-pathogenic interactome directly translates into eco-evolutionary dynamics along with niche colonisation generated with the help of mutations and gene ontology reports. Evolutionary game theory goes hand in hand with generating various niche colonisation reports based on evolutionary paradigms like ESS. Created with BioRender.com.

Eco-evolutionary feedback (EEF) hinges on two prerequisites: a population’s robust interaction with its environment and the ensuing evolutionary response within the population (Post and Palkovacs, 2009). This phenomenon of niche construction (NC) predominantly stems from the exoproducts and metabolic activities of the participating bacteria. For instance, the virulence assembly of *S. aureus* involves intricate binding proteins and coagulases, which not only modify the environment but also influence the dynamics of the microbial consortia. In contrast, *P. aeruginosa* deploys an array of exoproducts that bolster its survival by lysing and eliminating other bacteria (McNally and Brown, 2015). NC and niche segregation hold significant sway in the persistence of complex organisms and bacteria, shaping their traits through neighbor-dependent selection (Vasseur et al., 2011). Structured populations thriving in host-mimicking media play a pivotal role in the endurance of competing bacteria (Zhao et al., 2019). Interestingly, while *P. aeruginosa* eradicates *S. aureus* in most laboratory media; the elimination does not transpire in *in vivo* or wound-like media (WLM) (Kvich et al., 2022), underscoring the involvement of specific media components in sustaining this persistence. Thus, it partly elucidates why studying persistence is captivating and challenging compared to other microbial interactions. **Figure 3** provides an overview of the process of identifying persistence and attempting its analysis. The Allee effect introduces a trade-off between expansion (acute phase) and survival (Smith et al., 2014). An intriguing quantification metric could be absolute growth, which amalgamates growth with a bacteria’s metabolism (Pajon et al., 2023). This might offer a more accurate assessment for analysing polymicrobial infections, given their pronounced dependence on the environment.

EGT examines bacterial decisions within specific conditions without delving into their consequences. It also falls short in capturing the entirety of bacterial decisions across various situations. To address these limitations, researchers turn to eco-evolutionary dynamics, a robust approach for analysing interactions, which complements EGT. This gives rise to eco-evolutionary game theory (EEGT), an area that remains insufficiently explored. The concept delves into the mathematical representation of how the environment changes due to evolutionary shifts, a focal point of study since the concept (Tilman et al., 2020; Cao and Wu, 2021; Wang et al., 2023). Cao et al., demonstrate the coexistence of mutant and wild-type bacteria hinges on environmental degradation rates, notably elevated in bacteria-related scenarios. These rates surge with the progression of infections, particularly chronic ones, thereby fostering persistence. Thus, the framework proves valuable for understanding polymicrobial persistence, as exemplified by prevalent models like dental caries and chronic wounds (**Figure 3**).

## 8 Persistence in polymicrobial infections

Analyzing persistence is challenging due to its inherent evolutionary intricacies. The phenomenon arises when competing bacteria strike a balance to share a niche they collectively construct, as observed in concepts (Kassen et al., 2004; Mukherjee et al., 2023). The evolutionary principle applies to microbial persistence in chronic infections and within the resident microbiota. For instance, *Pseudomonas fluorescens* adapts to float in static cultures through variant development (Rainey and Travisano, 1998). Such trade-offs are complex and multi-faceted to analyze but are crucial to understanding. These adaptive mutants exhibit negative frequency dependence; a substantial surge in their population triggers self-regulation and decline.

Consequently, delving into persistence necessitates the study of variants, or mutants, emerging within populations. The key focus is their stable coexistence with the prevailing wild-type strains, a dynamic that EGT captures. The process of mutant creation orchestrated by the environment and its consequent impact on community ecology finds elucidation through the lens of eco-evolutionary dynamics.

Another intriguing facet of persistence unfolds when one bacterium undergoes modifications to align with the metabolic needs of another bacterium (Hansen et al., 2007). Hansen et al. illustrate the coexistence-driven growth of *Pseudomonas putida* alongside Acinetobacter spp., facilitated by their shared utilization of benzoate. Similarly, *P. aeruginosa’s* utilization of acetoin generated by *S. aureus* underscores the cooperative persistence achieved through syntropy (Camus et al., 2020). Moreover, evidence suggests that carbon catabolite repression potentially contributes to the synergistic relationship between *P. aeruginosa* and *S. aureus* in chronic wounds (Park et al., 2020). Catabolite repression’s impact is noteworthy, often leading to the emergence of loss-of-function and quorum-sensing mutants in *P. aeruginosa* by intricately connecting molecular proteins with mutant evolution (Mould et al., 2022). Intra-genic interactions also play a role, especially when bacteria share similar resource consumption rates.

An instance of this is the synergistic hemolysin production observed in *S. aureus* and *S. epidermidis*, influencing the availability of nutrients such as iron in the environment and subsequently reducing competition (Hébert and Hancock, 1985). A study showcasing *E. coli’s* coexistence via trade-offs through an acetate link adds weight to these observations (Mukherjee et al., 2023). It also, reinforces the concept of syntropy between *P. aeruginosa* and *S. aureus*, with EGT becoming established through metabolic trade-offs.

## 9 Polymicrobial niche colonization

Bacteria display remarkable versatility, employing strategies to enhance their fitness and acclimate to diverse environments, spanning from thermophiles (Lusk, 2019) to halophiles (Corral et al., 2019). The underlying eco-evolutionary dynamics play a critical role in understanding the trade-offs that bacteria undergo during this process. At the interface of host-pathogen interactions, host nutritional immunity and immune responses create the substantial potential for bacteria within chronic infections to undergo mutations, shaping their adaptation (Baishya and Wakeman, 2019).

Previous research indicates that within wound environments, anaerobic bacteria tend to inhabit the middle region, sustained by oxygen consumers (Choi et al., 2019). Compelling *in vivo* evidence supports the notion that *P. aeruginosa* predominantly colonizes the deeper layers of wounds, while *S. aureus* tends to establish itself on the wound’s surface (Fazli et al., 2009; Chen et al., 2021; Khalid et al., 2023). Pouget et al., showed aggregated biofilms of both *S. aureus* and *P. aeruginosa* at the specific locations still maintaining a mixed polymicrobial biofilm with enhanced characteristics (Pouget et al., 2022). Interestingly, studies propose that the chronicity of wounds is not significantly tied to biofilm formation. Instead, the importance of factors like Type III Secretion System (T3SS) and anaerobic genes emerge. It suggests that *P. aeruginosa* might exist in an anaerobic state within these contexts (Morgan et al., 2019).

To grasp the concept of persistence, it’s imperative to dissect the evolved niche. *S. aureus*, an integral component of the skin microbiome, serves as the primary colonizer (Byrd et al., 2018). Consequently, even before *P. aeruginosa* enters the wound, *S. aureus* has already initiated virulence production and biofilm formation. Drawing accurate conclusions necessitates *in vitro* experiments that avoid simultaneous inoculation of both bacteria or prevent initial *S. aureus* colonization. Such scenarios could yield misleading outcomes. When co-cultures of *P. aeruginosa* were introduced into pre-established biofilms of *S. aureus*, the growth of the former was significantly impaired, revealing a substantial fitness disadvantage (Tognon et al., 2017). Following the principles of the “war of attrition” from EGT, an optimal strategy involves a robust virulence response. Therefore, for *P. aeruginosa* to enhance its payoff, it must either elevate its competitive edge or increase its virulence. In anaerobic environments, *P. aeruginosa* undergoes mutations in the *wsp* signaling system, resulting in heightened quorum sensing and increased production of staphylocidal proteins (Tognon et al., 2017). Consequently, *P. aeruginosa* might actively seek anaerobic settings to boost its fitness in the face of competition, potentially leading to niche segregation.

According to the “tragedy of the commons” principle in EGT, the emergence of quorum sensing mutants is expected when quorum sensing is upregulated in *wsp* mutants. However, empirical evidence suggests that in co-cultures, quorum-sensing mutants struggle to survive due to the bacterium’s emphasis on maintaining fitness (Luján et al., 2022). Yet, as *P. aeruginosa* reaches deeper wound regions where its fitness is no longer influenced by *S. aureus*, the development of *lasR* mutants might occur. The communities structured as biofilms tend to foster opportunistic interactions, reflecting the applicability of the “tragedy of the commons” concept in infection scenarios where bacteria are in proximity (Hansen et al., 2007). This dynamic contributes to the stabilization of inter-specific communities. The anaerobic milieu aids the growth of oxygen-sensitive *lasR* mutants, stemming from interactions involving pyocyanin and molecular oxygen (Castañeda-Tamez et al., 2018) as well as Mhr, which captures O_2_ in microoxic conditions (Clay et al., 2020). This underscores the pivotal role of the environment in dictating infection persistence. These signal-blind mutants, termed “cheaters,” play a central role in intra-specific competition, aligning with the tenets of the tragedy of the Commons. In this context, intra-specific competition operates as a stabilizing mechanism within Eco-Evolutionary Dynamics (EED), fostering persistence.

Chen et al. conducted a study that illuminates how hyperglycemic conditions (levels exceeding 50mg/dL) lead to the downregulation of *P. aeruginosa* virulence, effectively demonstrating the direct impact of the environment in maintaining persistence (Chen et al., 2020). In another study, Sanchez et al. unveiled that the survival of social mutants is dependent upon the presence of eco-evolutionary feedback loops, underscoring the pivotal role the environment plays (Sanchez and Gore, 2013). Similarly, Smith et al. uncovered that limitations could arise from albumin sequestration of 3OC12-HSL, thereby diminishing the efficiency of the *las* quorum sensing system and consequently curtailing the production of its exoproducts. It highlights yet another facet of how the environment can influence bacterial dynamics (Smith et al., 2017).

**Figure 4:**
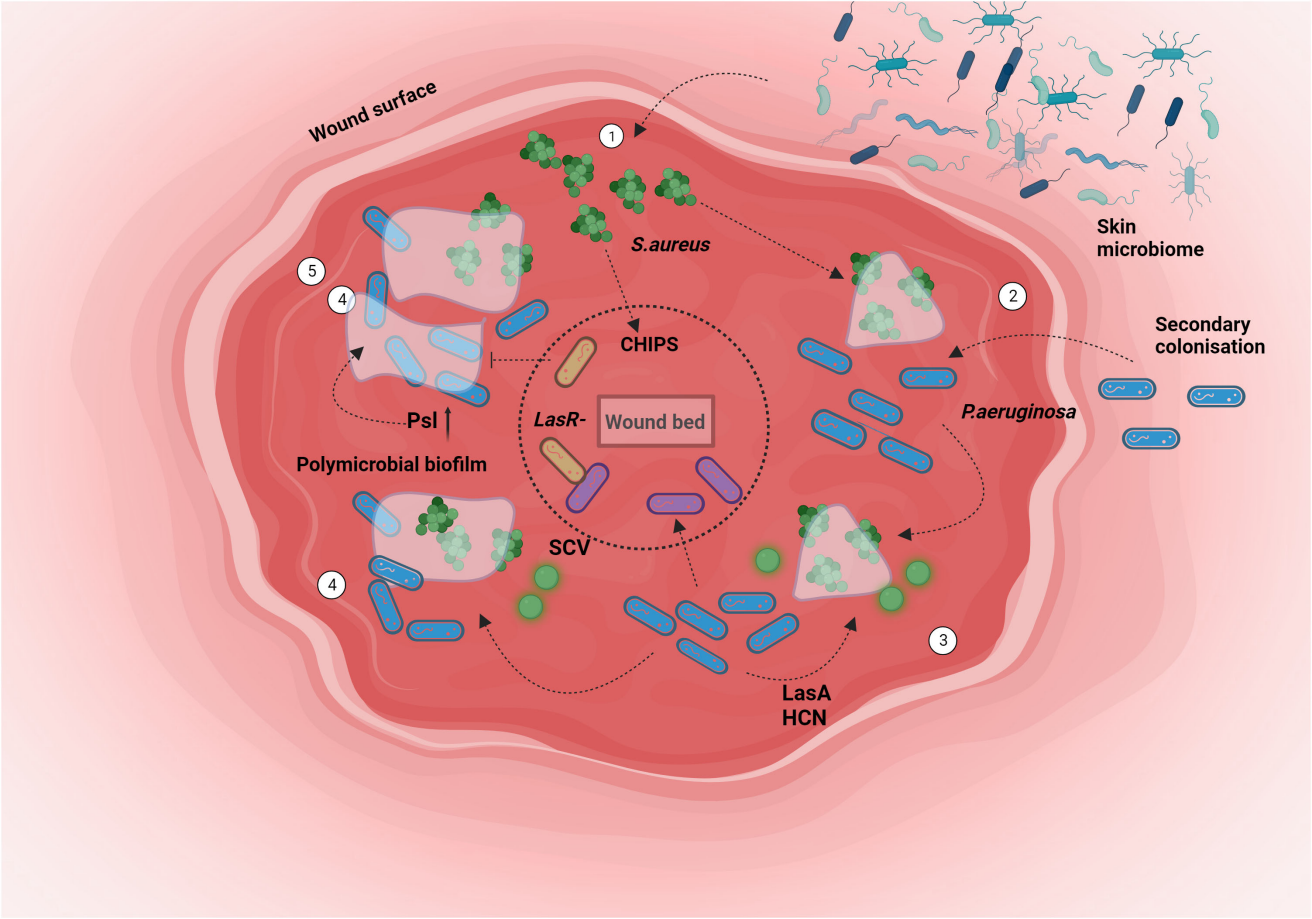
Chronic wound spatio-temporal colonisation: Bacterial niche colonization involves a complex interplay of host and pathogenic factors. In scenarios like chronic wounds, the formation of polymicrobial biofilms facilitates intricate interactions among various participants. The behavior of *P. aeruginosa* is intricately linked to the presence of the primary colonizer, *S. aureus*. The sequence unfolds as follows: 1. *S. aureus* initiates colonization of the wound from the skin. 2. *P. aeruginosa* enters the wound alongside the pre-established *S.aureus* population. 3. The exoproducts produced by *P. aeruginosa* prompt the emergence of a Small Colony Variant (SCV) variant of *S. aureus*. Subsequently, *P. aeruginosa* extends its presence to the deeper wound regions. 4. This progression culminates in the formation of a polymicrobial biofilm. 5. Niche segregation occurs, fostering a state of coexistence between both bacterial species. Created with BioRender.com.

The persistence of both *P. aeruginosa* and *S. aureus* has been linked to diminished quorum sensing effectiveness and a decline in the competitive edge of *P. aeruginosa* (Zhao et al., 2019). The viability of cheaters is additionally influenced by the longevity of the shared resource within the medium (Kümmerli and Brown, 2010). Smith et al. have further demonstrated that the presence of upregulated pyocyanin production in *lasR* mutants renders them unable to eliminate *S. aureus* (Smith et al., 2017). In situations of high relatedness, factors such as enhanced growth, immune suppression, and overall virulence can be favored, thereby promoting kin selection (West and Buckling, 2003) (**Figure 4**).

## 10 Applications of ED

Microbiology encompasses three significant dimensions: therapeutics, diagnostics, and fundamental science. A comprehensive understanding of these components is essential for advancing the field of microbiology. Further exploration into EEGT holds the potential to open novel frontiers across all three dimensions, propelling advancements in therapeutics, diagnostics, and fundamental scientific understanding.

### 10.1 Diagnostics

Diagnostics, a comprehensive field within microbiology, primarily revolves around identifying the microbial causes of diseases and characterizing various microbial properties. Common techniques used for diagnosis include culturing, quantitative polymerase chain reaction (qPCR), and microscopy. While these methods are considered the gold standard, they are often time-consuming and can sometimes yield misleading results. These methods are increasingly being replaced by Next Generation Sequencing (NGS) or Whole Genome Sequencing (WGS). NGS has already been successfully applied to clinical isolates (Hasman et al., 2014; Peterson et al., 2023). The major drawback of WGS is the expensive equipment and reagent costs. While these costs are decreasing with advancements, alternatives can also be considered (Muhamad Rizal et al., 2020). In recent times, biosensors have gained significant popularity as a versatile tool in the realm of diagnostics (Mehrotra, 2016; Metkar and Girigoswami, 2019; Saylan et al., 2021). Biosensors, that use a biosensing element to generate electrical signals. Biosensors offer broad screening capabilities, exhibit robust viability and stability, and can be easily manufactured on a large scale (Su et al., 2011).

Among these biosensors, bacto-sensors utilizing bacteria can be categorized into two main types: Microbial Surface Display Biosensors (MSDBs) and Microbial Whole Cell Biosensors (MWCBs). MSDBs utilize bacterial surface receptors to capture target analytes, distributing them within a matrix for subsequent analysis. On the other hand, MWCBs employ whole bacterial cells, often genetically modified, as miniature reactors to quantify or qualify specific metabolites (Gui et al., 2017). For instance, photogenic bacteria like *Photobacterium mandapamensis* have been employed to detect pathogens causing urinary tract infections (UTIs), such as *E. coli*, *Proteus mirabilis*, *S. aureus*, and *C. albicans* (Reyes et al., 2020). In cases where bacteria aren’t naturally photogenic, they can be engineered to express reporter genes like luciferase and green fluorescence protein, generating bioluminescence upon the activation of targeted genes (Martinez et al., 2019; Bazhenov et al., 2023). The disadvantages of this methodology are the low selectivity, viability, and durability. Long-term cultivation is still a hurdle faced by microbial biosensors. To address this, synthetic biology has introduced genetic circuits that enable cells to produce specific responses under defined conditions, working in tandem with bacto-sensors (Del Valle et al., 2021). For example, isogenic mutant bacteria have been instrumental in studying the virulence production of *S. typhimurium* (Gulig, 1993). To enhance their fitness, mutated bacteria with synthetic circuits are subjected to compensatory evolution after being grown in specific media, although this approach can sometimes result in circuit breakdown (Levin et al., 2000; Yang et al., 2020; La Rosa et al., 2021). An innovative solution to this problem involves leveraging the concept of the rock-paper-scissors game, ensuring remarkable stability for maintaining synthetic circuits (Liao et al., 2019). Bacto-sensors have been effectively employed to detect pathogenic markers in clinical samples (Chang et al., 2021). Moreover, advanced biosensors capable of dynamically tailoring treatments based on *in vivo* conditions with dynamic control have also been successfully utilized (Vaaben et al., 2022).

### 10.2 Basic science

Fundamental scientific understanding forms the bedrock of various critical biological phenomena. It stands as the foundational pillar for the two interconnected branches of microbiology: diagnostics and therapeutics. In recent times, there has been a significant surge in microbiome research, with the gut microbiome being a prominent focus. Within this realm, game theory has proven instrumental in dissecting the nutritional utilization of gut bacteria, yielding insights into the development of our gut microbiome (Angell and Rudi, 2020). A captivating observation highlights a stag game-like interaction within the gut microbiome, where *Firmicutes* and *Bacteroidetes* collaborate to counteract an invader, *Actinomyces* (Li and Ma, 2020). This cooperative guild of *Firmicutes* and *Bacteroidetes* showcases positive interactions that extend across various microbiomes resident in the host. The *Firmicutes*-to-*Bacteroidetes* (F: B) ratio emerges as a noteworthy biomarker, pivotal in assessing a range of dysbiosis conditions such as diarrhea and irritable bowel syndrome. Moreover, this ratio’s implications extend to well-known effects on conditions like obesity, type 2 diabetes, and atherosclerosis (Magne et al., 2020). This interplay mirrors the Stag game in evolutionary game theory, where individuals must decide between hunting alone (the evolutionarily stable strategy) or collaborating in a group while considering the risks of uncooperative behaviour (**Figure 1**) (Luo et al., 2021). Commensal bacteria employ diverse mechanisms to counteract pathogenic invaders, encompassing colonization resistance (Sorbara and Pamer, 2019), immune modulation (Lin et al., 2021), and chemical secretions (Teng et al., 2023). A crucial aspect of this dynamic ecosystem lies in bacterial vesicles, which exert influence on the gut-brain axis and facilitate inter-kingdom communication, underscoring the significance of eco-evolutionary dynamics (Pirolli et al., 2021). Similar dynamics can be observed in interactions between *Candida albicans* and *Staphylococcus aureus* against *Pseudomonas aeruginosa*. While both *C. albicans* and *S. aureus* individually aid each other’s fitness, they unite against the common threat posed by *P. aeruginosa.* Remarkably, both *C. albicans* and *S. aureus* are integral components of the skin’s normal microbiota. It is imperative to recognize the intricate interplay among these microorganisms and their implications. This underscores the essence of basic science in microbiology, shaping our comprehension of microbial dynamics and their broader impacts on health and disease (**Table 1**).

### 10.3 Therapeutics

Therapeutics existing against polymicrobial infections are very minimal. This is because of the aforementioned drastic shift in metabolic, phenotypic and resistance profiles of the various bacteria involved. This is further complicated by the need to target specific GC bacteria which are pivotal for the pathogenesis. Currently two gold standards have been used without involving eco-evo manipulations. Antimicrobial peptides (AMP’s) and photodynamic therapy have been recently gaining traction among researchers involved in polymicrobial research.

AMPs are a class of proteins that can be engineered to form specifically targeted antimicrobial peptides (STAMPs) (Batoni et al., 2016; Manzo et al., 2019). AMPs are a powerful choice, but they do pose multiple limitations such as possibility of protein degradation, sequestration, inactivation by ionic salts and potential toxicity to eukaryotic cells (Batoni et al., 2021). The drawbacks of AMPs are fixed by photodynamic therapy. Photodynamic therapy involves the usage of photosensitizers that are capable of producing ROS under illumination of light (Martins Antunes de Melo et al., 2021). This ensures a drastic decrease in the bacterial quantity and can also be manipulated to help in visualization of such polymicrobial biofilms (Li and Wu, 2022). These photosensitizers can be altered to reach a certain location but their activity cannot be limited to killing specific bacteria, further their activity necessitates the need for oxygen and appropriate microenvironment (Karner et al., 2020). This is hence a huge drawback for PDT as it doesn’t allow specific targeting within a biofilm. While both methods are tried and tested against polymicrobial biofilms there needs to be a middle ground. The application of evolutionary dynamics can enable us to reach this fairly stable middle ground.

**Figure 5:**
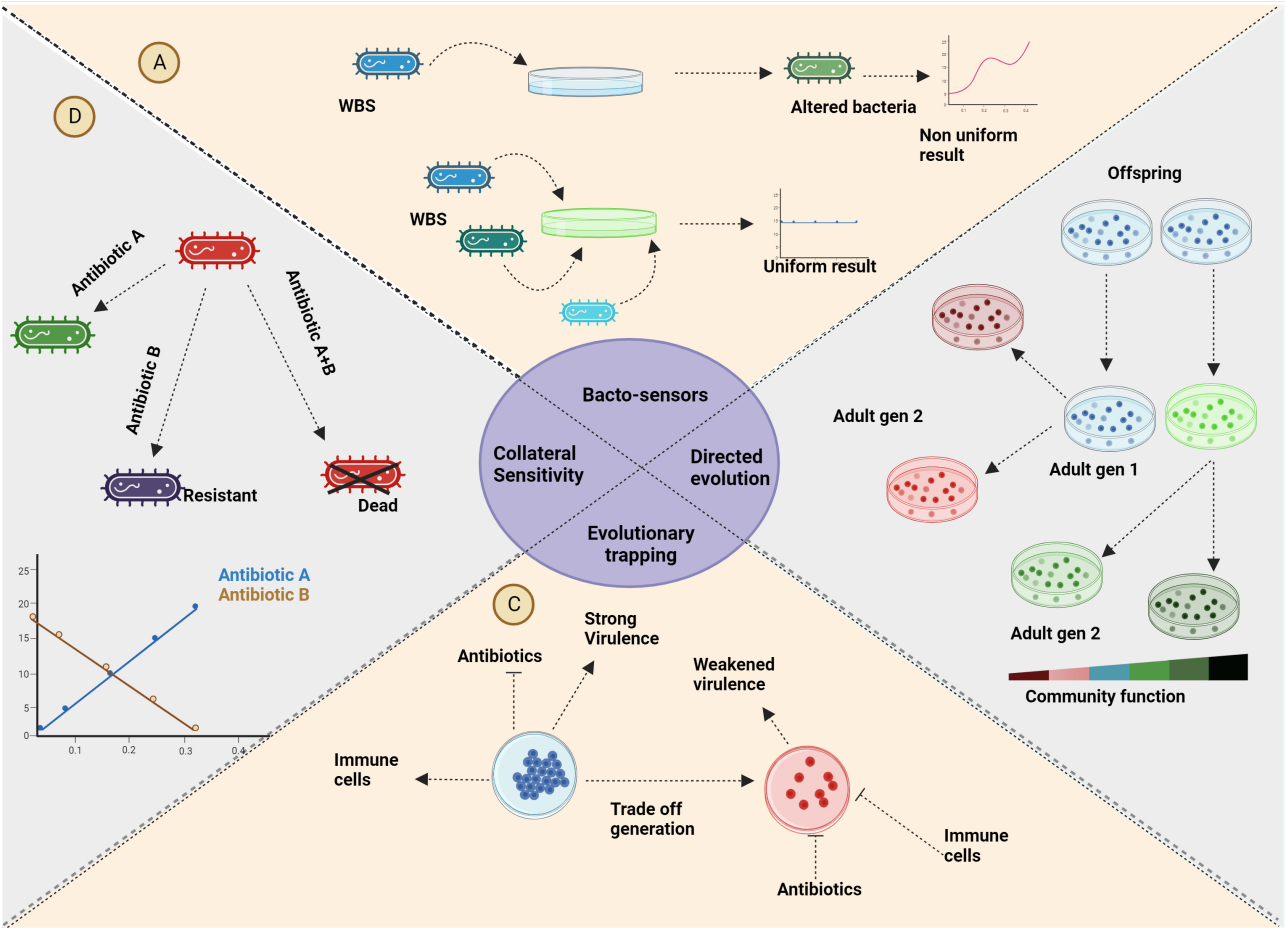
Applications: **(A)** The utilization of the rock-paper-scissors game extends to the improvement of real-time analysis for genetically modified bacterial sensors. **(B)** Reducing inter-specific competition through selective processes yields bacteria with enhanced community functions. Employing eco-evolutionary dynamics guides bacterial evolution toward desired functionalities. **(C)** As a strategy against antimicrobial resistance (AMR), evolutionary trapping introduces trade-offs in bacteria that render them vulnerable to host defences or antibiotics. The vaccine usually given alters the environment altering the eco-evolutionary dynamics involved in favour of medication/immunity. **(D)** Similar to evolutionary trapping, collateral sensitivity can be harnessed to address infections involving antibiotic-resistant strains. Bacteria that acquire resistance against a specific antibiotic through a trade-off will gain sensitivity collaterally to another antibiotic. Created with BioRender.com.

A direct application of the principles underlying eco-evolutionary dynamics is the creation of what are known as “evolutionary traps.” Diard and colleagues demonstrated this concept by developing a vaccine that prompts Salmonella bacteria to mutate, causing them to lower their protective sugar coat, rendering them susceptible to the immune system (Diard et al., 2021). This process is achieved through directed evolution or artificial selection, where bacteria are selected from an initially high-functioning community, cultivated, and iteratively refined to enhance community functioning. The above approach can also be achieved through niche modulation, gaining similar effects (Andrei et al., 2019). On the same lines, a very common evolutionary trap is through collateral sensitivity. Bacteria that turn resistant to one antibiotic become susceptible to another, which is of immense clinical significance (Roemhild and Andersson, 2021). This trade-off in resistance can be harnessed by targeting bacterial metabolism, providing a potential avenue for tackling antimicrobial resistance (AMR). This innovative approach is explored in-depth in a review by Sanz-García and colleagues (Sanz-García et al., 2023). Furthermore, the principles of evolutionary game theory have given rise to adaptive therapy, which has demonstrated the capacity to influence the eco-evolutionary dynamics of cancer. This strategy aims to induce collateral sensitivity within cancer cells, thereby guiding the course of the disease. These examples highlight the profound interconnections between various applications spanning diverse domains within microbiology (Acar et al., 2020). In essence, the utilization of eco-evolutionary dynamics has led to the development of strategies such as evolutionary traps and collateral sensitivity, effectively addressing challenges in fields ranging from microbial infections to cancer therapy. These applications underscore the depth of integration between seemingly disparate areas within microbiology (**Figure 5**).

## 11 Prospects and discussion

The complexity inherent in polymicrobial infections makes their treatment challenging. The dynamics of such infections mirror the rock-paper-scissors game, resulting in a “survival of the weakest” scenario, underscoring the need for meticulous treatment approaches. Addressing the intricacies of these infections requires a comprehensive perspective. To this end, a zone model was devised to study polymicrobial wounds, highlighting the importance of investigating the exobiofilm-rich zone 3, characterized by virulence and host enzymes. (Kirketerp‐Møller et al., 2020). The intricate protein-protein interactome assumes a pivotal role in comprehending and managing polymicrobial infections. Gaining insight into persistence necessitates an exploration of polymicrobial adaptive radiation, aiming to comprehend the diverse selection pressures at play during infection. Focused research is essential to discern the impact of allelic polymorphism, intra-species, and inter-species heterogeneity on virulence factors within polymicrobial consortia (Azimi et al., 2020). The phenomenon of kin recognition in bacteria warrants exploration to grasp the mechanisms of cheater control across bacterial species (Wall, 2016). Furthermore, developing multitrophic models is imperative to gain a deeper understanding of coexistence among multiple competing bacteria. Both theoretical and empirical investigations within the realm of eco-evolutionary game theory are needed, especially to analyze complex “N” player games. The extensive applications of evolutionary game theory and eco-evolutionary dynamics, as discussed, must be reframed through the lens of extended eco-evolutionary game theory to generate multifaceted and beneficial strategies (Levine et al., 2017). Recently Skwara et al. showed a highly novel method of analysing community function using statistics. They generated the functional landscape using the generalized Lotka-Volterra model (gLV), a prominent model in predator-prey dynamics in eco-evolutionary dynamics (Skwara et al., 2023). To effectively address these infections, research should encompass zone analysis, protein interactions, adaptive radiation, allelic polymorphism, kin recognition, and multitrophic models.

## Author contributions

ASr: Conceptualization, Investigation, Writing – original draft. ASa: Conceptualization, Investigation, Writing – original draft. SR: Writing – review & editing. HD: Writing – review & editing. APS: Conceptualization, Investigation, Supervision, Writing – review & editing.
